# The Meiotic Nuclear Lamina Regulates Chromosome Dynamics and Promotes Efficient Homologous Recombination in the Mouse

**DOI:** 10.1371/journal.pgen.1003261

**Published:** 2013-01-31

**Authors:** Jana Link, Daniel Jahn, Johannes Schmitt, Eva Göb, Johannes Baar, Sagrario Ortega, Ricardo Benavente, Manfred Alsheimer

**Affiliations:** 1 Department of Cell and Developmental Biology, Biocenter, University of Würzburg, Würzburg, Germany; 2 Biotechnology Program, CNIO, Madrid, Spain; Harvard Medical School, United States of America

## Abstract

The nuclear lamina is the structural scaffold of the nuclear envelope and is well known for its central role in nuclear organization and maintaining nuclear stability and shape. In the past, a number of severe human disorders have been identified to be associated with mutations in lamins. Extensive research on this topic has provided novel important clues about nuclear lamina function. These studies have contributed to the knowledge that the lamina constitutes a complex multifunctional platform combining both structural and regulatory functions. Here, we report that, in addition to the previously demonstrated significance for somatic cell differentiation and maintenance, the nuclear lamina is also an essential determinant for germ cell development. Both male and female mice lacking the short meiosis-specific A-type lamin C2 have a severely defective meiosis, which at least in the male results in infertility. Detailed analysis revealed that lamin C2 is required for telomere-driven dynamic repositioning of meiotic chromosomes. Loss of lamin C2 affects precise synapsis of the homologs and interferes with meiotic double-strand break repair. Taken together, our data explain how the nuclear lamina contributes to meiotic chromosome behaviour and accurate genome haploidization on a mechanistic level.

## Author Summary

Diploid germ line cells have to undergo meiosis to produce haploid gametes. Haploidization involves pairing and recombination of homologous chromosomes as a prerequisite for their proper segregation. Pairing of homologous chromosomes requires their active repositioning within meiotic nuclei, which depends on the interaction of telomeres with the nuclear envelope. This dynamic association is vital for a faithful meiosis and thus crucial for fertility. However, very little is known about the relationship between telomeres and nuclear envelope components. Here, we have investigated the role of the nuclear lamina, a structural scaffold that is intimately associated with the inner nuclear membrane. In somatic cells, the lamina is a key player in chromatin organization and fulfils various functions such as nuclear structure maintenance and regulation of transcription. In order to understand its role in meiosis, we investigated lamin C2, the only A-type lamin isoform expressed in mammalian meiotic cells. We demonstrate that lamin C2 is essential for timely repositioning of meiotic telomeres. In its absence, synapsis of homologous chromosomes and double-strand break repair are severely affected. These multiple meiotic defects lead to infertility in males. We conclude that the nuclear lamina contributes directly to fertility through facilitating meiotic chromosome movements.

## Introduction

Correct segregation of the chromosomes during meiosis depends on accurate prearrangement of the homologs that culminates in their precise and unambiguous pairing. Recent studies established that the nuclear envelope (NE) plays an important role during these processes. It functions as a platform for telomere driven chromosome rearrangement, which is essential for chromosome pairing and synapsis [1]. This special role requires a general reorganization of the NE which also involves the nuclear lamina, a structural protein network underlying the inner nuclear membrane (INM). Through its multiple interactions with a variety of proteins, the lamina functions in nuclear organization and maintenance as well as regulation of transcription [2]. Because of the many regulatory and structural roles, impaired lamina function is responsible for numerous severe human diseases, collectively termed laminopathies, which are often caused by mutations within the *LMNA* gene that codes for the A-type lamin proteins [3], [4].

Mammalian meiotic cells are distinguished by the absence of three of the four lamin isoforms that are typically expressed in differentiated somatic cells [5]. Instead, they express, together with lamin B1, a unique lamin, lamin C2, which is a short meiosis-specific A-type lamin isoform encoded by the *LMNA* gene [6], [7]. Compared to its somatic counterparts, meiotic lamin C2 is an N-terminally truncated version which lacks the complete N-terminal head including a substantial part of the rod domain. As a consequence the structure differs from that typically observed in somatic lamins [8], [9]. This N-terminal truncation is quite remarkable, as it concerns protein domains, which have been shown to be crucial for the assembly into higher order structures [10], [11]. Thus, lamin C2 resembles a “natural deletion mutant” that features unique properties and, consistent with this, shows altered polymerization and higher mobility compared to other A-type lamin isoforms [12]. Strikingly, the distribution of lamin C2 in meiotic cells differs significantly from the typical patterns shown for lamins in somatic cells. While somatic lamins usually distribute evenly along the NE, lamin C2 forms distinct domains within the nuclear lamina of meiocytes [13]. In early prophase I, telomeres attach to and subsequently move along the inner nuclear membrane [14]. During these movements, attached telomeres are permanently embedded within the lamin C2 enriched domains. Therefore, it was suggested that lamin C2 locally modulates the NE to allow proper telomere attachment and/or movement [13].

A first indication for such a role came from a previous study investigating spermatogenesis in *Lmna* ^−/−^ mice that were supposed to lack all A-type lamin isoforms [15], [16]. In that study, A-type lamins emerged to be essential for male fertility. Furthermore, the obtained results indicated that the general integrity of A-type lamin expression is critical for correct synapsis of homologous chromosomes in male meiocytes [16]. However, *Lmna* ^−/−^ mice actually lack expression of both meiosis-specific lamin C2 and somatic A-type lamins A and C and, as a consequence, show a strong somatic disease phenotype. This matter sets obvious limitations to the interpretation of the results obtained in the *Lmna* ^−/−^ genetic background. For example, in the given genetic background one cannot exclude possible side effects that might arise from defective somatic cells of the male gonad. Therefore, it is not clear whether the observed meiotic phenotype is caused by the absence of meiotic lamin C2 or is rather a result of a more general lamin A/C dependent somatic cell dysfunction [16]. It should be noted, that we recently demonstrated that the *Lmna* ^−/−^ mice aberrantly express an, as yet unrecognized, short progerin-like A-type lamin, a matter which further complicates the interpretation of the former results obtained in the *Lmna* ^−/−^ background [17]. So far, no additional, detailed analysis of the role of A-type lamins for meiotic homologous pairing and recombination in females has been carried out. Hence, the molecular mechanism by which lamins could contribute to homologous pairing and promote normal meiotic progression has remained elusive.

To address these issues, we for the present study decided to generate a lamin C2 isoform specific knockout model that, hence in a clear-cut genetic background, allows analysing the direct impact of lamin C2 on meiotic events.

## Results/Discussion

**Figure 1:**
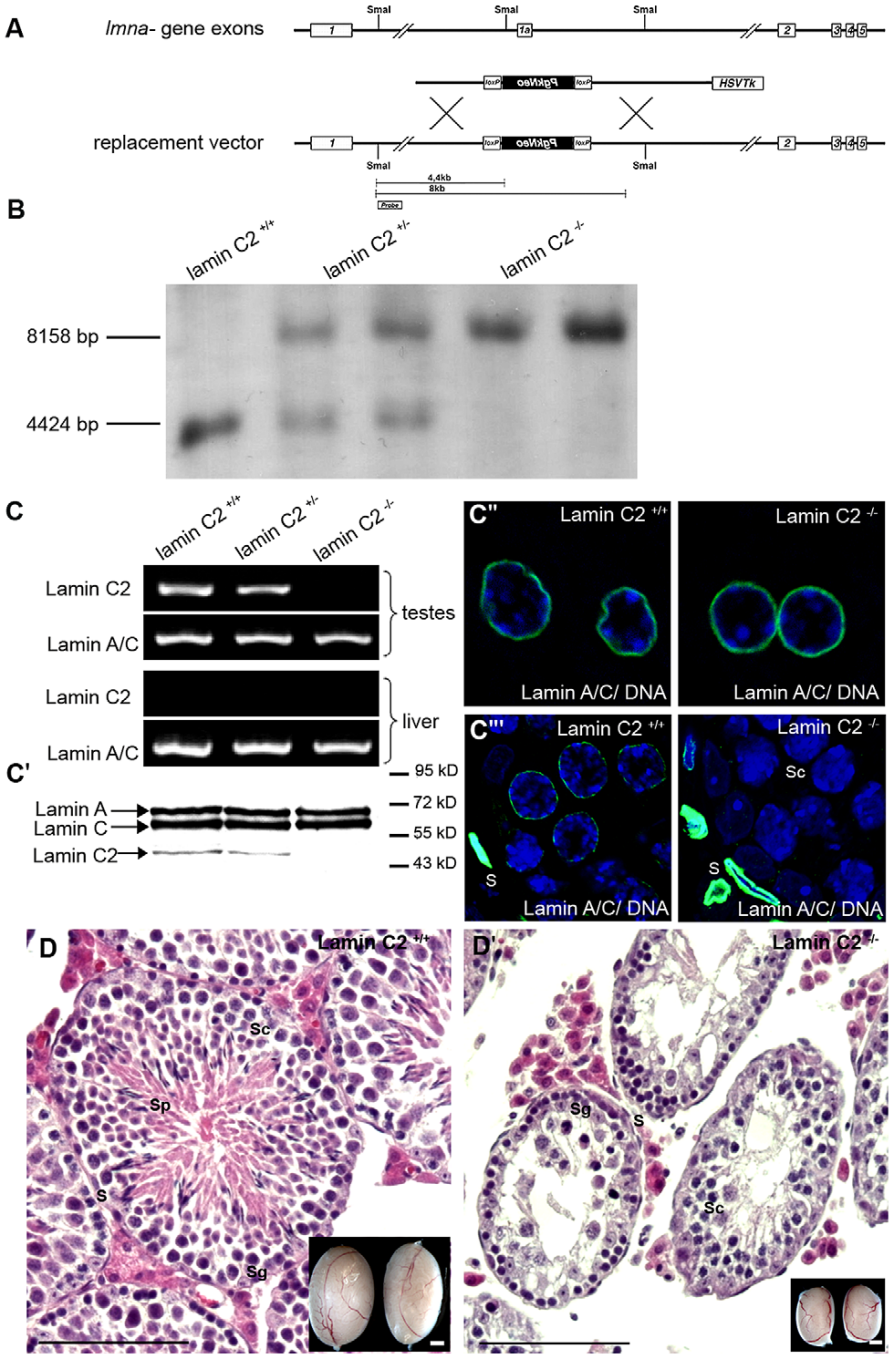
Generation and characterization of a lamin C2-deficient mouse line. (A) Murine somatic lamins A and C are composed of exons 1 through 12 and 1 through 10 of the *Lmna* gene, respectively, both excluding exon 1a. Lamin C2 is encoded by exons 1a through 10 with exon 1a serving as an alternative starting exon specific for lamin C2 [18]. Exon 1a was targeted for construction of a knockout mouse model deficient for lamin C2. SmaI restriction sites and the probe used for southern blot analyses are indicated. (B) Southern blot analyses of genomic mouse DNA digested by SmaI distinguishing wildtype, heterozygous and homozygous lamin C2-deficient animals. (C, C′) RT-PCR and immunoblot analyses showing presence of lamin C2 in the testes of wildtype and heterozygous mice, but absence in *lamin C2^−/−^* testes: expression of lamin A/C remained unaffected in all three genetic backgrounds. (C″, C′″) Immunohistochemical analyses using anti-A-type lamin antibodies on liver and testis tissues showing unaffected expression of A-type lamins in somatic cells, while in meiotic cells of *lamin C2^−/−^* testes A-type lamins are completely absent. (D) Histological sections of wildtype and *lamin C2^−/−^* testes demonstrating the absence of post-meiotic cells in *lamin C2^−/−^* tissue. Scale bars 100 µm. As shown in the insets, testes size of *lamin C2^−/−^* males is significantly reduced. Scale bars 1 mm. S somatic cells, Sg spermatogonia, Sc spermatocytes, Sp spermatids.

We created a targeting construct to selectively eliminate the lamin C2 specific exon 1a, while all other regions of the *Lmna* gene were left intact to ensure regular expression of the somatic A-type lamin isoforms (Figure 1; see also [18]). Successful targeting was verified through various approaches (Figure 1). Correct deletion of the lamin C2 specific exon 1a in heterozygous and homozygous animals was confirmed by Southern blot analysis (Figure 1B). As expected, subsequent RT-PCR and Western blot analysis demonstrated that both lamin C2 mRNA and protein are present in the testes of wildtype males, but are clearly absent from the testes of *lamin C2^−/−^* mice. Further immunohistochemical approaches using anti-A-type lamin antibodies confirmed that *lamin C2^−/−^* male meiocytes fail to express any A-type lamin. Co-testing for somatic A-type lamin isoforms revealed that somatic lamin A/C expression is virtually not affected by the deletion of the lamin C2 specific exon 1a as in wildtype, heterozygous and *lamin C2^−/−^* mice comparable amounts of lamins A and C could be detected in liver cells as well as in somatic cells of the testes (Figure 1C). These results are fully consistent with previous reports demonstrating that lamin C2 is the only A-type lamin expressed in meiocytes [7]. More importantly, they also confirm that the applied exon specific targeting strategy selectively disrupted the expression of meiosis specific lamin C2, but not of somatic lamins A and C.

Detailed analysis of the phenotype revealed that, in clear contrast to the previously described *Lmna^−/−^* mice which show severe laminopathy-associated somatic tissue dysfunction [15], [16], mice deficient for lamin C2 were fully viable and of normal size and weight. Repeated mating attempts of *lamin C2^−/−^* males with wildtype females never produced offspring implying that *lamin C2^−/−^* males were completely infertile. Lamin C2-deficient females, however, produced offspring when mated with wildtype males, indicating a sexual dimorphic impact of lamin C2 on fertility (see below). These results are consistent with the histological appearance of the gonads. As in the wildtype, ovaries from 11 days postpartum (dpp) and 28 dpp lamin C2-deficient females contained growing diplotene oocytes (data not shown). *Lamin C2^−/−^* males, however, had significantly smaller testes compared to wildtype controls (Figure 1D). Detailed histological analyses of testes from adult *lamin C2^−/−^* mice revealed that post-meiotic stages were completely absent from the seminiferous tubules (Figure 1D). Consequently, also no sperm were found within the epididymis (Figure S1). TUNEL assay on *lamin C2^−/−^* testes sections revealed a high frequency of cell death in regions of the seminiferous tubules, where prophase I stages predominated (Figure S1), indicating that mutant spermatocytes are unable to complete meiosis and are removed by apoptosis.

**Figure 2:**
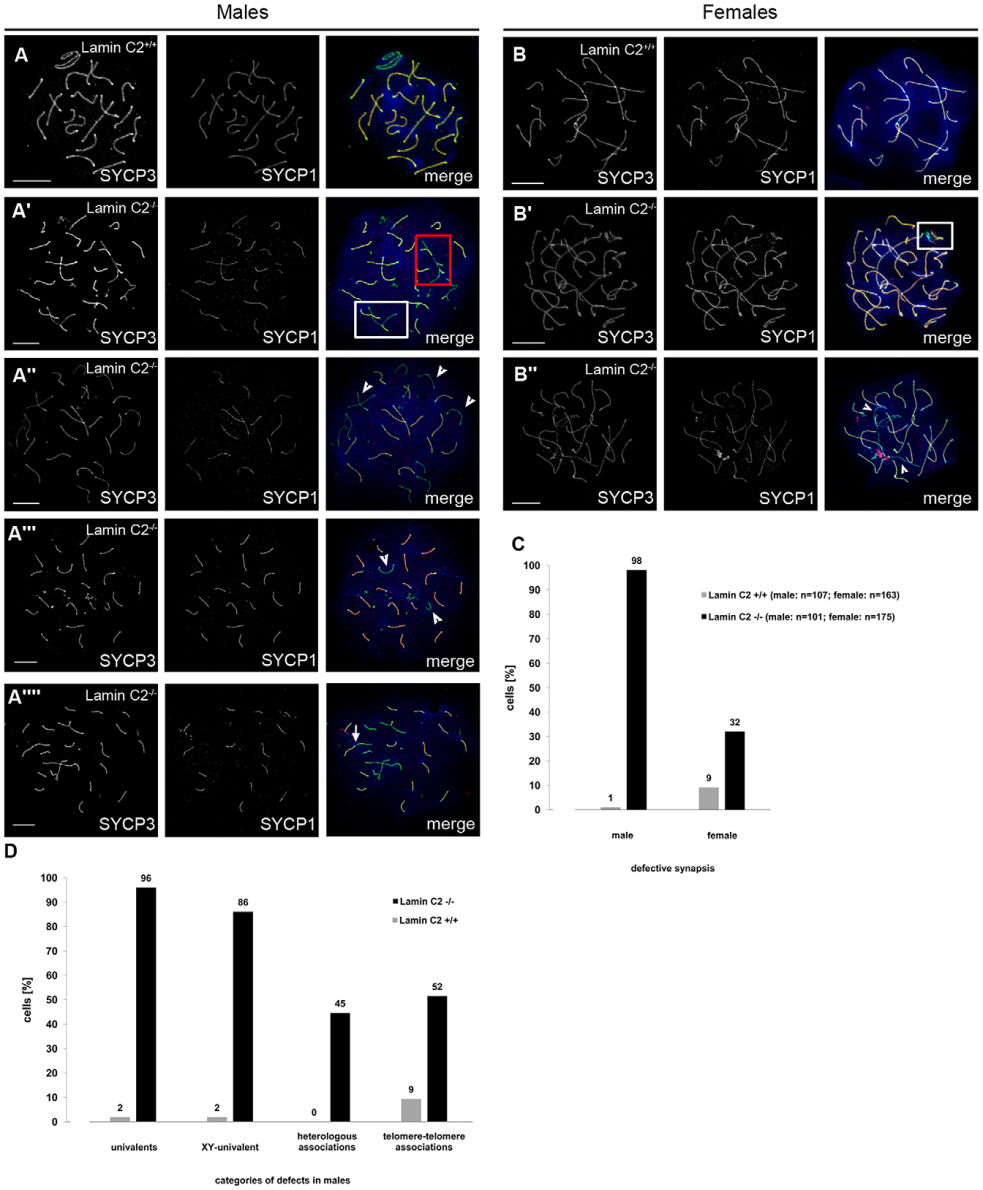
Defective synapsis of the homologous chromosomes in *lamin C2^−/−^* meiocytes. Representative chromosome spreads of spermatocytes (A) and oocytes (17.5 dpf) (B) labelled with anti-SYCP3 and anti-SYCP1 antibodies. Heterologous associations (red box in A′) are observed in *lamin C2^−/−^* spermatocytes only. Incomplete pairing of homologs (white boxes in A′ and B′) as well as univalent chromosomes (arrowheads in A″,A′″ and B″) occur in both *lamin C2^−/−^* spermatocytes and oocytes. *Lamin C2^−/−^* spermatocytes also display cells with X and Y chromosomes as the only univalents (arrowheads in A′″) or with linear telomere-telomere associations between non-homologous chromosomes (arrow in A″″). Scale bars 10 µm. (C) Quantification of meiocytes with defective synapsis in *lamin C2^−/−^* males and females. For both sexes, differences between mutants and controls are highly significant (Pearson's Chi^2^ test p-value<0.0001 each) (D) Synaptic pairing defects in males were further categorised and quantified. Interestingly, sex chromosomes were univalent in the vast majority of mutant spermatocytes. See text for further discussion.

To investigate the actual function of lamin C2 during gametogenesis in closer detail, we analysed the consequences of its absence on meiotic progression. A key feature of meiosis and an indispensable requirement for correct genome haploidization is the precise and unambiguous pairing of the homologs and their subsequent physical linkage (synapsis) mediated by the synaptonemal complex. Chromosome spread preparations of *lamin C2^−/−^* pachytene meiocytes revealed frequent defects in synaptic pairing of the homologs in both sexes, although sex-specific differences were observed (Figure 2A–2C). In males, quantification of pairing defects (Figure 2D) showed that virtually none (<1%) of the *lamin C2^−/−^* spermatocytes showed normal meiotic progression, while in wildtype controls >98% of pachytene spermatocytes appeared normal. Univalent chromosomes were clearly the most frequent defect observed, as 96% of mutant spermatocytes showed varying numbers of unsynapsed chromosomes in cells, where fully synapsed homologs were also present (Figure 2A″). Prominently, the sex chromosomes, where synapsis is reduced to the pseudoautosomal region, remained univalent in 86% of *lamin C2^−/−^* spermatocytes (Figure 2A′″). Further frequently observed phenomena in knockout spermatocytes were heterologous associations between non-homologous chromosomes and associations between telomeres (in 45% and 52%, respectively; Figure 2A′, 2A″″). Analysis of lamin C2-deficient females disclosed that synapsis of the homologs was also affected in oocytes, though in a less dramatic manner. While most wildtype mid-pachytene oocytes isolated from 17.5 days post fertilisation (dpf) embryos achieved full synapsis (91%), a significant portion (32%) of lamin C2-deficient oocytes showed defective synapsis that, in contrast to the situation in males, most frequently manifested as pairs of homologs that initiated, but did not complete synapsis (Figure 2B′). In order to exclude a simple delay in synapsis formation at mid-pachytene stage, we also analysed synapsis defects in late-pachytene oocytes (19.5 dpf). In the absence of lamin C2 we found a significantly increased number of late-pachytene oocytes having overt synaptic defects (wt: 13.33%, n = 75; *lamin C2^−/−^*: 27.38%, n = 84; Pearson's Chi^2^ test p-value 0.033) which is similar to the situation found in mid-pachynema. However, consistent with the pronounced differences in phenotypes of males and females, we found an increased average number of chromosomes affected by synaptic defects per cell in males compared to females (Figure S2). Overall, these results demonstrate that loss of lamin C2 significantly interferes with chromosome synapsis in mammalian meiocytes of both genders with sex-specific differences regarding the severity of meiotic complications.

Telomere driven formation and release of the meiotic bouquet at the leptotene/zygotene stage is a well-conserved phenomenon that has been shown to be essential for preparing later events of meiosis [1], [14], [19]–[23]. Current models suggest that bouquet formation enhances homologous pairing by increasing proximity of homologous chromosomes. Furthermore, the release of the bouquet conformation may be a means of preventing incorrect associations of non-homologous chromosomes [19]. In recent years, it has been established that meiotic tethering and moving of telomeres within the nuclear envelope (NE) depend on SUN- and KASH-proteins [24], [25] and that this is broadly conserved. These form LINC-complexes, thereby creating a connection between nuclear and cytoskeletal components [26]. For mammals, an involvement of SUN1 and SUN2 in NE attachment of meiotic telomeres has been reported [27], [28]. Moreover, impairment of telomere attachment has repeatedly been shown to cause chromosome synapsis defects and thus interferes with correct progression of mammalian meiosis [28]–[30]. Nonetheless, the mechanisms by which meiotic telomeres are attached and repositioned have remained largely unclear. Particularly, direct functional evidence for an involvement of nuclear lamins in these processes is missing.

**Figure 3:**
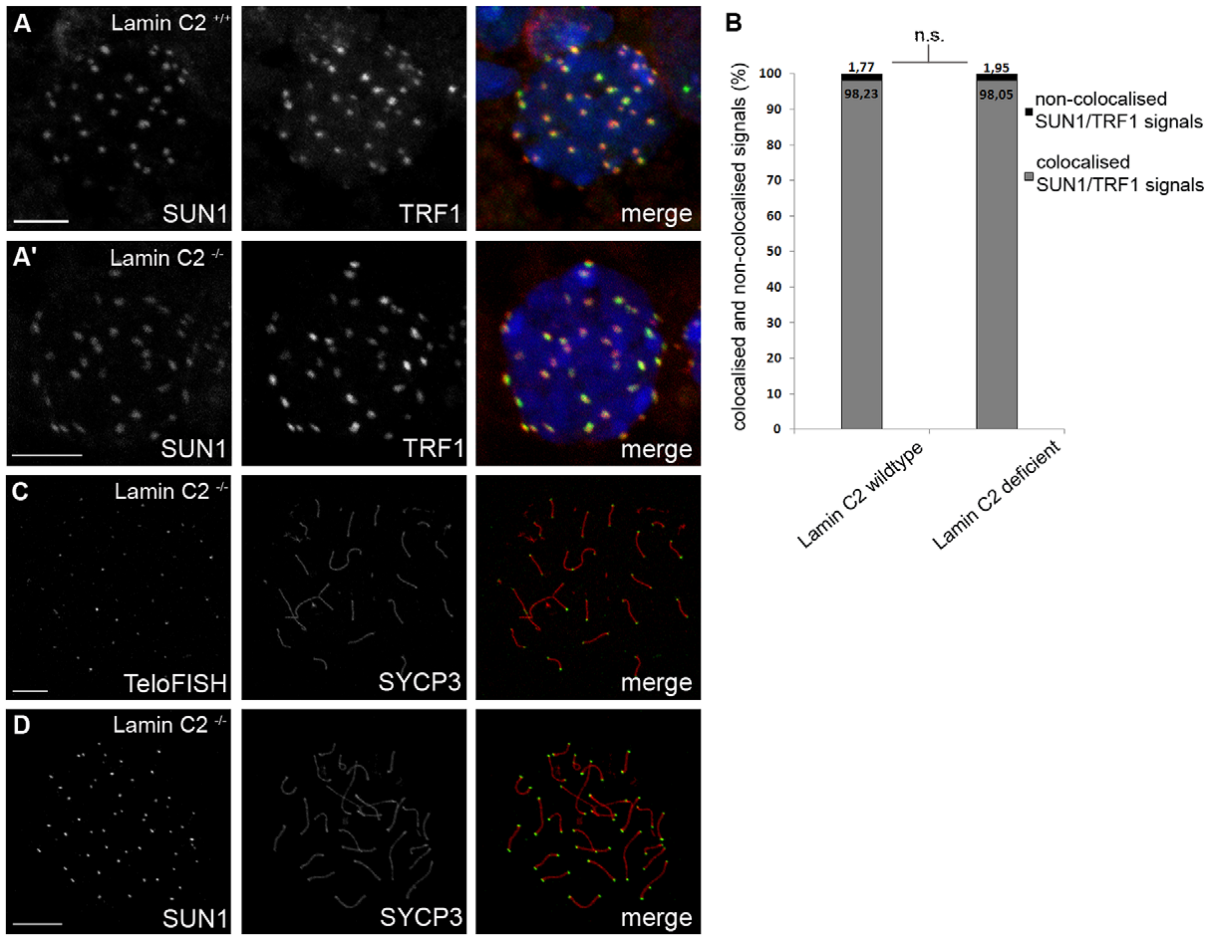
Loss of lamin C2 has no effect on meiotic telomere attachment. (A) 3D-preserved swab preparations showing wildtype (A) and knockout (A′) spermatocytes simultaneously labelled with anti-TRF1 and SUN1 antibodies. As in the wildtype, in *lamin C2^−/−^* spermatocytes virtual all telomeres appear to be attached to the NE as indicated by co-localisation of TRF1 and SUN1 signals. Scale bars 5 µm. (B) Quantifications of co-localised and non-co-localised TRF1/SUN1 signals (see A) revealed that ratios of co-localised to non-co-localised spots comparing wildtype and knockout spermatocytes show no significant difference (wildtype *n* = 33; *lamin C2^−/−^ n* = 45; Pearson's Chi^2^ test p-value: 0.799). (C,D) Chromosome spread preparations of pachytene-like *lamin C2^−/−^* spermatocytes showing that all telomeres are associated with SUN1. In (C) TeloFISH and in (D) anti-SUN1 staining in co-localisation with SYCP3. Scale bars 10 µm.

Since lamin C2 is enriched at the sites of telomere attachment [13], an obvious reason for synaptic defects and meiotic disruption as seen in *lamin C2^−/−^* males could be impairment of telomere attachment. To address this issue, we used SUN1, an NE protein known to tether meiotic telomeres [28], [29], in co-localisation experiments with fluorescently labelled telomeres to quantify telomere attachment in *lamin C2^−/−^* spermatocytes [30] (Figure 3A). Quantifying co-localised and non-co-localised telomere and SUN1 signals revealed no statistically significant difference between wildtype and knockout spermatocytes (Figure 3B, p-value: 0.799 using Pearson's Chi^2^ test). Consistent with this, virtually all telomeres were located at the nuclear periphery in 3D reconstructed nuclei of mutant spermatocytes (Video S1). Furthermore, chromosome spreads of pachytene-like *lamin C2^−/−^* spermatocytes demonstrated that, in fact, all telomeres were connected to SUN1 as all chromosome axes had SUN1 foci on both ends (Figure 3C, 3D). This clearly shows that, even though telomeres are embedded within lamin C2 enriched domains, lamin C2 is dispensable for telomere attachment to the NE.

**Figure 4:**
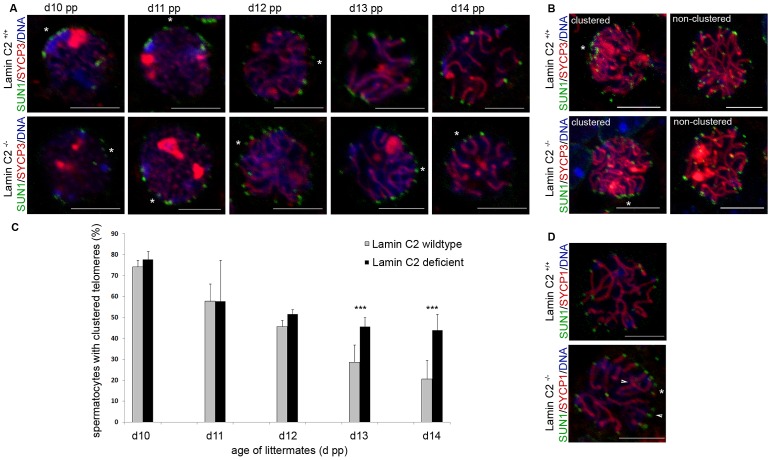
Bouquet release is significantly delayed in *lamin C2^−/−^* spermatocytes. (A) Representative wildtype and knockout spermatocytes from testes of 10 to 14 day old mice labelled for SYCP3 and SUN1 with clusters of telomeres indicated by asterisks. Scale bars 5 µm. (B) Z-projections of entire nuclei from wildtype and knockout littermates, labelled again for SYCP3 and SUN1, demonstrating the distinguishable difference between clustered and non-clustered telomere patterns; asterisks indicate telomere clusters. Scale bar 5 µm. (C) Quantitative evaluation of spermatocytes showing clustered telomeres in wildtype and knockout littermates aged 10 to 14 dpp. Note the highly significant differences at ages 13 and 14 dpp. Values represent means (± s.d.) of data from three littermate pairs of wildtype and knockout animals each, separately analysed using Pearson's Chi^2^ tests: d10 *p = *0.28 (wt, *n = *517; *lamin C2^−/−^*, *n = *580); d11 *p = *0.15 (wt, *n = *677; *lamin C2^−/−^*, *n = *703); d12 *p = *0.29 (wt, *n = *736; *lamin C2^−/−^*, *n = *780); d13 *p = 6.5 x10^−3^* (wt, *n = *639; *lamin C2^−/−^*, *n = *648); d14 *p = 7.6 x10^−7^* (wt, *n = *700; *lamin C2^−/−^*, *n = *682). ***p<0.01 (D) Representative wildtype and *lamin C2^−/−^* spermatocytes from day 14 aged mice showing comparable stages of synaptonemal complex assembly as indicated by SYCP1 labelling. Arrowheads denote homologous pairing defects in the absence of lamin C2; the asterisks indicate telomere clustering. Scale bar 5 µm.

As telomere-driven meiotic chromosome rearrangement, which lead to bouquet conformation and its subsequent release, are prerequisites for intact synapsis formation [31], we asked whether lamin C2 has a role in movement rather than in attachment of telomeres. In order to address this question, we analysed the temporal behaviour of telomere movements during bouquet formation and release over the first wave of spermatogenesis. Quantification of 3D reconstructed spermatocytes from testes tissue of sequential ages (10 to 14 dpp) with regard to their state of telomere clustering (Figure 4, Video S2) revealed a distinct bouquet resolution phenotype caused by lamin C2 deficiency. In the wildtype, at 10 dpp, when most cells synchronously reach leptotene/zygotene transition [32], 74.2% of spermatocytes were in the bouquet stage showing a typically clustered telomere pattern (Figure 4A, 4C). Compared to the wildtype situation, at 10 dpp *lamin C2^−/−^* mice showed no significant alterations in bouquet frequency (77.6%). Similarly, at 11 dpp there was no difference in bouquet frequency between wildtype and knockout siblings, both showing 57.8% of spermatocytes with clustered telomere patterns. Hence, spermatocytes lacking lamin C2 appear to have no problem in attaining bouquet stage. With progression of spermatogenesis bouquet configuration is resolved. Accordingly, in wildtype testes we found gradually decreasing numbers of bouquet stages with 45.4%, 28.7% and 20.5% at 12, 13 and 14 dpp, respectively. In corresponding *lamin C2^−/−^* littermates, however, numbers of spermatocytes in bouquet stage remained significantly elevated. In particular, knockout animals showed 51.8%, 45.4% and 43.9% of spermatocytes with clustered telomeres at ages of 12, 13 and 14 dpp, respectively. Compared to their wildtype siblings at 13 and 14 dpp, knockout mice roughly showed the 1.5 fold and 2.5 fold amount of bouquet stages, resulting in statistically highly significant differences at these ages (mean p-values for 13 and 14 dpp<0.01 using Pearson's Chi^2^ test; Figure 4C). Nonetheless, wildtype and knockout animals reached comparable sub-stages of prophase I at 14 dpp as judged by progression of synaptonemal complex assembly (Figure 4D). Thus, our results demonstrate that while NE attachment *per se* is not affected, the movement of telomeres during bouquet release is significantly delayed in male mice lacking lamin C2. Since release of the bouquet is thought to promote resolution of incorrect chromosomal associations [31], impairment of timely bouquet resolution appears to be the basic mechanism responsible for the meiotic defects observed in lamin C2-deficient mice, i.e. defective synapsis formation.

Consistent with the findings reported by other groups, our observations suggest a similar dependency of efficient homologous pairing on telomere clustering and movement in mammals as has been previously described for yeast. In fission yeast it has been shown, that telomere clustering and repositioning is required for efficient chromosome alignment and subsequent association [33], [34]. Within this line of argument, studies in budding yeast have discussed the roles of Ndj1 and Csm4 in meiotic telomere dynamics [35], [36]. In *csm4* mutants, where meiotic telomeres are still associated with the NE, the absence of telomere-led chromosome movements, rather than altered DSB repair, serves as an explanation for the observed homolog non-disjunction. Because of the previously determined molecular properties of lamin C2 and its ability to alter NE integrity [12], we conclude that lamin C2 locally modulates NE properties at the sites of telomere attachment to allow efficient directed telomere movement and thus promotes homologous chromosome synapsis. Lack of lamin C2 in turn could reduce local NE flexibility and, by this means, interfere with regular movement of attached telomeres as found here in the lamin C2-deficient background.

**Figure 5:**
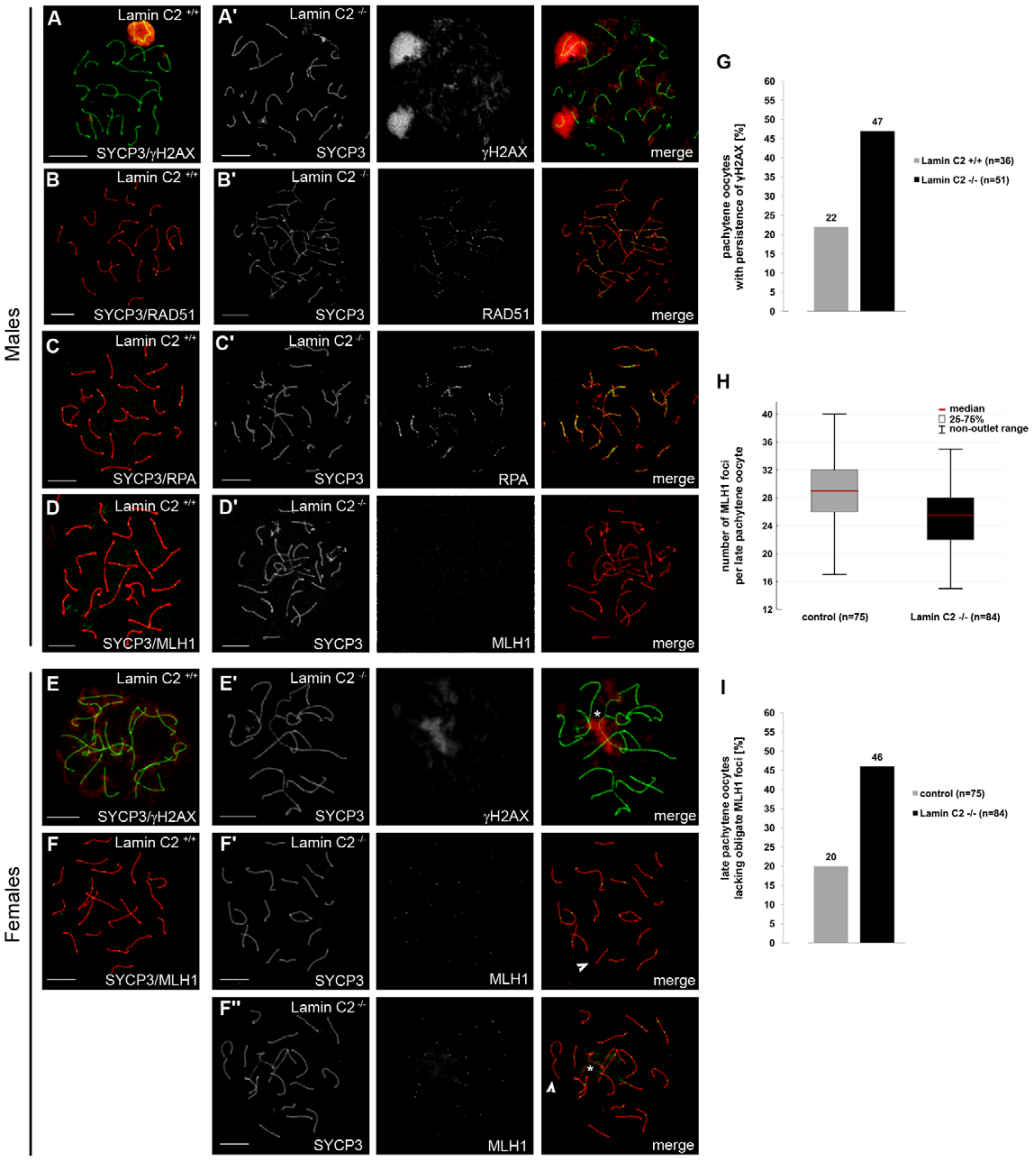
Alterations of meiotic DNA double-strand break (DSB) repair and inefficient homologous recombination caused by lamin C2 deficiency. Chromosome spread preparations were double-stained using anti-SYCP3 antibodies in combination with anti-γH2AX (A,E), anti-RAD51 (B), anti-RPA (C) or anti-MLH1 (D,F) antibodies. (A–D) In males, *lamin C2^−/−^* spermatocytes show incomplete repair of induced DSB as demonstrated by the increased persistence of γH2AX (A′), RAD51 (B′) and RPA (C′) and they lack crossing over events indicated by the complete absence of MLH1 (D′). (E) In females, efficient DSB repair is also affected in lamin C2-deficient pachytene oocytes (17.5 dpf) as revealed by intensive γH2AX staining surrounding incompletely synapsed chromatin (asterisk in E′). (F) In contrast to males, *lamin C2^−/−^* females are able to recruit MLH1 onto chromosome axes in 19.5 dpf late-pachytene oocytes. However, the MLH1 foci number was lowered and a significant portion of cells lacked at least one obligate MLH1 focus indicating the absence of cross over recombination on the affected chromosomes (arrowheads in F′ and F″). This phenomenon was likewise seen in oocytes with (asterisk in F″) or without (F′) obvious synaptic defects. Scale bars 10 µm. (G) Quantification of pachytene oocytes with persistence of γH2AX in wildtype and knockout littermates reveals inefficient DSB repair in lamin C2-deficient cells (Pearson's Chi^2^ test p-value = 0.0323). (H) Whisker box plot showing the number of MLH1 foci per cell in late pachytene oocytes of *lamin C2^−/−^* and control (*lamin C2^+/+^* and *lamin C2^+−/^*) animals. Two pairs of littermates were analysed. Knockout oocytes show a significant reduction of MLH1 foci number, indicating a lowered rate of homologous recombination (median foci numbers: control, 29; *lamin C2^−/−^*, 25.5; Mann-Whitney U-test p-value = 0.00025). (I) The number of late pachytene oocytes lacking one or more obligate MLH1 foci is significantly elevated in *lamin C2^−/−^* animals compared to the controls (*lamin C2^+/+^* and *lamin C2^+/−^*). As above, two pairs of littermates were analysed (Pearson's Chi^2^ test p-value = 0.0005).

Since chromosome synapsis, homologous recombination and bouquet formation and release are closely interdependent processes during mammalian meiosis [37], [38], we then asked whether loss of lamin C2 has a direct effect on recombination as well. To assess lamin C2 function in recombination we next examined selected markers of DSB repair. In early meiotic prophase, sites of DSBs, introduced by SPO11, become strongly labelled by γH2AX, a phosphorylated H2A histone variant associated with unrepaired DSBs. During leptonema and zygonema it is found in large domains around the DSBs. As meiotic prophase I progresses, γH2AX labelling successively disappears from the autosomes and, in the male, becomes restricted to the sex chromosomes [39] (Figure 5A). In pachytene-like *lamin C2^−/−^* spermatocytes, however, γH2AX remained associated with most of the chromosomes in a cloud-like manner, indicating that meiotic DSBs are formed, but are not efficiently repaired. Interestingly, sex chromosomes, although they often failed to synapse in knockout spermatocytes, showed strongly γH2AX labelled chromatin (Figure 5A′). Further analysis of later stages of DSB processing revealed that in lamin C2-deficient pachytene-like spermatocytes numerous RAD51 and RPA signals, which mark early and intermediate stages of DSB repair [37], aberrantly persist along both paired and unpaired axes (Figure 5B′, 5C′). In wildtype late pachytene spermatocytes MLH1, a component of late recombination nodules and a marker of presumed crossing overs [40], appeared with at least one distinct MLH1 focus per pair of synapsed homologs. These foci were consistently absent from paired and unpaired chromosome axes of *lamin C2^−/−^* spermatocytes (Figure 5D, 5D′). Overall, this indicates that recombination events are initiated in lamin C2-deficient spermatocytes, but repair is not efficient or complete and functional crossing overs do not form. Nonetheless, the formation of sex body specific chromatin does occur even if synapsis between sex chromosomes is defective. This affirms that *lamin C2^−/−^* spermatocytes initiate, but fail to complete, pachynema, a phenomenon observed in numerous knockout models of meiosis-specific proteins [41]. Inducing apoptosis during mid-pachynema of spermatocytes carrying defects, such as incomplete synapsis, points to an activation of a pachytene-checkpoint mechanism preventing defective germ cells from further maturation [42].

Aberrant persistence of RAD51 and RPA on chromosome axes and the observed lack of MLH1 in lamin C2-deficient spermatocytes might be a consequence of the activation of the male pachytene-checkpoint and the subsequent elimination by apoptosis in mid-pachynema rather than a direct effect of lamin C2-deficiency on maturation and completion of homologous recombination *per se*. Mammalian oogenesis apparently lacks a stringent checkpoint operating at mid-pachytene stage and, thus, allows for analysis of recombination in late stages of meiotic prophase I [43]. Hence, we performed a detailed examination of recombination in *lamin C2^−/−^* oocytes at 19.5 dpf. Consistent with the above described findings, lamin C2-deficient oocytes of 19.5 dpf embryos reached late pachynema despite the persistence of synaptic defects (Figure 5F″). Additionally, temporal progression through meiotic prophase I stages *per se* appeared normal as, when compared to the wildtype, the ratio of pachytene to diplotene oocytes was not significantly altered in *lamin C2^−/−^* ovaries (n>50 each; Pearson's Chi^2^ test p = 0.5478). As in the males, γH2AX staining persisted until mid-pachytene stage, thereby surrounding incompletely synapsed chromosomes in *lamin C2^−/−^* oocytes from 17.5 dpf embryos (Figure 5E, 5G). In contrast to males however, lamin C2-deficient oocytes from 19.5 dpf embryos were able to recruit MLH1 onto chromosome axes at late pachynema (Figure 5F). Strikingly, quantification of MLH1, revealed a significant reduction of MLH1 foci in *lamin C2^−/−^* females, indicating a reduced rate of meiotic recombination (Figure 5H). Moreover, while 80% of wildtype late pachytene oocytes had at least one obligate MLH1 focus on each bivalent, 46% of lamin C2-deficient oocytes completely failed to recruit MLH1 at least on one pair of homologs, regardless whether or not they exhibited synaptic defects (Figure 5I). Absence of MLH1-marked recombination nodules indicates the lack of cross over recombination on the affected chromosomes that, in the female, may account for increased chromosome segregation defects at later stages of meiosis [40], [44], [45]. Notably, as shown here, *lamin C2^−/−^* females are fertile despite the fact that *lamin C2^−/−^* oocytes revealed overt defects in homologous recombination and chiasmata formation. To some extent this matter resembles the situation described for *Sycp3^−/−^* mice that show a severely disrupted synapsis and reduced chiasmata formation. Similar to the *lamin C2^−/−^* mice presented here, males deficient for SYCP3 are completely infertile. In female *Sycp3^−/−^* mice, by contrast, oocytes form chiasmata, but at a lower level than in the wildtype, which results in a reduction, but not a complete loss, of fertility [46].

Regardless of the sexual dimorphic impact on fertility, our analyses clearly demonstrated that the meiotic nuclear lamina has a central role in regulating chromosome bouquet dynamics and is therefore essential for correct progression of meiotic homologous recombination in both male and female mice. Such interdependencies between bouquet stage resolution and DSB repair and homologous recombination have been described earlier. Mice that are unable to induce meiotic DSBs due to the absence of SPO11 and those showing defects in late recombination events, as is the case in the *Mlh1^−/−^* background, also show elevated bouquet frequencies. However, in mice with altered early recombination phenotypes, caused by the lack of recombination proteins DMC1 or HOP2, no significant increase in bouquet stages could be observed [38]. This suggests, that whilst defects in the formation of DSBs during leptonema or alterations of late recombination events during late pachynema may influence bouquet duration, the intermediate steps of DSB repair and early recombination do not. In *lamin C2^−/−^* spermatocytes DSBs are definitely induced but the stages when late recombination events normally occur are not reached due to checkpoint induced apoptosis during mid-pachynema. Therefore, neither altered early DSB repair nor defective late recombination is able to explain the observed delay in bouquet stage release in *lamin C2^−/−^* spermatocytes. Since defects in earlier DSB repair and recombination do not influence bouquet stage frequencies, inefficient DSB repair in *lamin C2^−/−^* meiocytes is likely to be a consequence of the delay in bouquet stage release, rather than the converse. Consistent with this, loss of telomere-led dynamics also affects recombination and crossing over in budding yeast meiosis [35]. Though to some respect the effects of altered meiotic chromosome movements on crossing over events differ between the budding yeast mutants and our lamin C2-deficient mouse, the significance of directed telomere-led chromosome dynamics *per se* for homolog recombination and disjunction seem to be as widely conserved as the bouquet formation itself.

## Materials and Methods

### Ethics statement

All animal care and experiments were conducted in accordance with the guidelines provided by the German Animal Welfare Act (German Ministry of Agriculture, Health and Economic Cooperation). Mouse generation, housing, breeding and experimental protocols at the CNIO, Madrid, were performed in accordance with protocols revised and approved by the Institutional Ethics Committees of the CNIO and following the European Regulation (2010/63/UE of September 22, 2010). Animal housing and breeding at the University of Wuerzburg was approved by the regulatory agency of the city of Würzburg (Reference ABD/OA/Tr; according to §11/1 No. 1 of the German Animal Welfare Act). All aspects of the mouse work were carried out following strict guidelines to insure careful, consistent and ethical handling of mice.

### Generation of lamin C2 knockout mice

To generate a lamin C2 specific knockout mouse line, in which the expression of other A-type lamins is left intact, a replacement vector was constructed to selectively eliminate lamin C2 specific exon 1a and the flanking putative upstream promoter elements. Therefore, a 4 kb genomic region including exon 1a was replaced by a neomycin cassette in reverse orientation using a modified pKSloxPNT vector [47], [48]. The vector for homologous recombination was designed as follows (see also Figure 1A): a 1.9 kb genomic fragment located 1.5 kb upstream of exon 1a (amplified from mouse genomic DNA by PCR; oligonucleotides Table S1) was cloned into the SalI/KpnI restriction sites downstream of the neomycin cassette and a corresponding 5 kb fragment 2.5 kb downstream of exon 1a (oligonucleotides Table S1) was ligated into the EcoRI restriction site between the thymidine kinase and neomycin cassette. The replacement vector, linearized with KpnI, was electroporated into mouse R1 ES cells and recombinant clones were selected in the presence of G418 and gancyclovir as previously described [47]. Positively targeted ES clones were identified by PCR using external primers (oligonucleotide sequence for genotyping: Table S1) and correct targeting was confirmed by Southern blot. For Southern blot analysis 15 µg NsiI (not shown) or SmaI digested DNA derived from ES cells (or tail tips of *lamin C2^+/+^*, *lamin C2^+/−^* and *lamin C2^−/−^* mice) was separated on a 0.8% agarose gel, subsequently transferred to a nylon membrane and correct targeting was tested with both external (see Figure 1A) and neomycin probes. Blastocyst injection of one of the positive ES clones gave rise to germline transmitting chimeras that were mated to produce heterozygous founder mice. Intercrossing of *lamin C2^+/−^* founder mice produced offspring with all genotypes in mendelian ratio. To confirm the genotypes we performed RT-PCR, Southern blot and immunofluorescence analysis as described below.

### Tissue preparations

Gonads from wildtype, heterozygous and *lamin C2^−/−^* mice were either fixed and embedded in paraffin wax for sectioning, frozen in 2-methylbutane for swab preparations or freshly used for chromosome spreads. Gonads for paraffin embedding were fixed in either 1% PBS-buffered formaldehyde (pH 7.4) for 3 hours or in 4% overnight. Tissues were then dehydrated in an increasing ethanol series and infiltrated with paraffin at 58°C overnight. Tissue samples for swab preparations [49] were placed in 2-methylbutane at −70°C immediately after dissection. Procedures for chromosome spreads were adapted from de Boer et al. [50]. For this, fresh tissue samples were incubated in hypotonic buffer (30 mM TrisHCl, 17 mM Na-citrate, 5 mM EDTA, 50 mM sucrose, 5 mM DTT; pH 8.2). For spermatocyte spreads, testes tubules taken from the hypotonic buffer were resuspended in 20 µl of sucrose solution (100 mM) and transferred to a slide covered with 1% formaldehyde solution (1% formaldehyde, 0.15% Triton X-100; adjusted with NaOH to pH 9.2). Slides were incubated in closed moisture chambers for 2 h, followed by 30 min incubation with the lid left ajar; finally slides were dried in the opened chambers. For oocyte spreads, ovaries were transferred from hypotonic buffer to a small droplet of sucrose solution (50 µl) placed on a slide. Ovaries were then decapsulated, fragmented with forceps and incubated with gentle shaking for 10 min to elute oocytes. Debris was removed from the slides and an equal amount (50 µl) of 2% formaldehyde solution (2% formaldehyde, 0.15% Triton X-100; adjusted with NaOH to pH 9.0) was added to the droplet of sucrose containing the oocytes. Slides were then incubated in closed moisture chambers for 1 h, followed by a 30 min incubation with opened chambers at room temperature. Finally, slides were dried at 37°C for approximately 2 h.

### Histology

Standard histology was performed on 5 µm sections of paraffin-embedded tissues fixed overnight in 4% formaldehyde as described previously [16]. To visualize and identify apoptotic cells, TUNEL assays were carried out on 10 µm sections of paraffin embedded testes using the ApopTag Fluorescein In Situ Apoptosis Detection Kit (Millipore, Schwalbach, Germany) according to the manufacturer's protocol.

### Expression analysis by RT–PCR

To show absence of lamin C2 expression in *lamin C2^−/−^* mice and to verify that expression of somatic lamins A/C is not affected by lamin C2 isoform specific targeting, we performed RT-PCR analysis on wildtype, heterozygous and knockout mice. Total RNA was isolated from testes suspensions or liver tissues of six week old littermates. RNA isolation was performed using TriFAST™ (Peqlab, Erlangen, Germany) according to the manufacturer's manual. 1 µg of total RNA was used for reverse transcription using oligo(dT) primer and M-MLV reverse transcriptase (Promega, Mannheim, Germany). Using 1 µl of RT-reaction we performed PCRs specifically amplifying either lamins A/C or lamin C2 transcripts. In case of lamins A/C, transcripts were amplified using a 5′ primer corresponding to the ATG region of the lamin A/C specific exon 1, whereas for lamin C2 we used a 5′ primer selectively binding to the ATG region of the lamin C2 specific exon 1a. For both transcripts the same 3′ primer was chosen from a region shared by both the lamins A/C and the lamin C2 transcripts (see Table S1 for oligonucleotide sequences).

### Antibodies

Primary antibodies used in this study were: rabbit anti-lamin A/C (H-110; Santa Cruz, Heidelberg, Germany), rabbit anti-SYCP3 (anti-Scp3; Novus biologicals, Littleton, CO), guinea pig anti-SYCP3, rabbit anti-SYCP1, guinea pig anti-SYCP1 [51], rabbit anti-TRF1 (TRF12-A; Alpha diagnostics, San Antonio, TX), mouse anti-γH2AX (Millipore), mouse anti-RPA (clone RPA34-20; Calbiochem, Darmstadt, Germany), mouse anti-MLH1 (clone G168-15; BD Biosciences, Heidelberg, Germany), rabbit anti-RAD51 (Calbiochem, Darmstadt, Germany) and guinea pig anti-SUN1 [52]. The corresponding secondary antibodies conjugated to Cy2, Texas red, Alexa647 (immunofluorescence) or peroxidase (immunoblot) were obtained from Dianova (Hamburg, Germany). Additionally, for FISH analysis a monoclonal mouse anti-digoxigenin and an anti-digoxigenin fluorescein conjugated fab fragment (Roche, Mannheim, Germany) were used according to the manufacturer's protocol.

### Expression analysis by SDS-PAGE and Western blotting

To assess the efficient disruption of lamin C2 and unaffected expression of lamins A/C in *lamin C2^−/−^* mice on the protein level, western blots using cell suspensions from whole testes preparations and from liver tissue (as a somatic control) of six week old wildtype, heterozygous and *lamin C2^−/−^* littermates were performed. Tissues or cells were resuspended in 2× SDS sample buffer (120 mM Tris/HCl, 10% SDS, 20% glycerine, 20% 2-mercaptoethanol; pH 6.8) and denatured at 95°C for 15 min before applying 5×10^5^ cells for each tissue onto a 12% SDS PAGE. After separation of the proteins with SDS-PAGE, proteins were transferred to a nitrocellulose membrane. Membranes were blocked overnight in TBST (10 mM Tris/HCl, 150 mM NaCl, 0.1%Tween 20) containing 10% milk powder. Anti-lamin A/C primary antibodies were diluted (1∶2000) in blocking solution and membranes were incubated for 60 min at room temperature with subsequent washing in TBST. Peroxidase-conjugated secondary antibodies were applied as specified by the manufacturer. Bound antibodies were detected using the Western Lightning Plus-ECL Enhanced Chemiluminescence Substrate (Perkin Elmer, Rodgau, Germany).

### Immunohistochemistry

Immunofluorescence analysis was carried out on chromosome spreads, swab preparations from frozen tissue or paraffin sections. Immunofluorescence staining of chromosome spreads were performed according to procedures adapted from de Boer et al. [50]. Blocking of cell spreads was performed with the supernatant of centrifuged (16.000 g, 30 min) blocking solution (5% milk, 5% FCS, 1 mM PMSF in DMSO; pH 7.4 in PBS). For double-label immunofluorescence, spreads were then incubated with the first primary antibody followed by washing in PBS before blocking again in blocking solution and incubating with the first secondary antibody. After another blocking step, slides were incubated with the second primary antibody followed by washing, reblocking and incubation with the second secondary antibody. For immunofluorescences on swab preparations, cells were fixed in PBS containing 1% formaldehyde for 10 min followed by permeabilisation in PBS/0.05% Triton X-100 for another 10 minutes. After washing and blocking in blocking solution, slides were incubated with both primary antibodies. Following this, slides were washed and blocked again and subjected to both secondary antibodies. To prepare paraffin sections for immunofluorescence, antigen retrieval and removal of paraffin was conducted as described before [53]. After washing the sections in PBS, sections were blocked with PBT (0.15% BSA, 0.1% Tween 20 in PBS, pH 7.4) prior to the incubation with both primary antibodies. After washing in PBS, sections were subjected to corresponding secondary antibodies. For all preparations DNA was counterstained using Hoechst 33258 (Sigma-Aldrich, Munich, Germany).

### Telomere fluorescence in situ hybridisation (TeloFISH)

To directly label telomeres, we performed fluorescence in situ hybridisation using digoxigenin-labelled (TTAGGG)_7_/(CCCTAA)_7_ oligomeres. After initial immunofluorescence staining (see above), spreads were subsequently briefly refixed for 20 min using 4% formaldehyde in PBS and washed in PBS. After rinsing slides in 2× SSC (0.3 M NaCl, 0.03 Na-citrate; pH 7.4) for 5 min, spreads were incubated in RNase A (100 µg/ml in 2× SSC) at 37°C for 1 hour. After rinsing in 2× SSC, cells were denatured for 20 min at 95°C in the presence of 10 pmol of each labelled probe in 40 µl of hybridisation solution (30% formamid, 10% dextrane sulphate, 250 µg/ml *E.coli* DNA in 2× SSC). Hybridisation was performed overnight at 37°C. After washing twice in 2× SSC at 37°C for 10 min, samples were blocked using 0.5% blocking-reagent (Roche) in TBS (150 mM NaCl, 10 mM Tris/HCl; pH 7.4). Probes were incubated for 1 h with anti-digoxigenin antibodies (Roche). After washing slides in TBST (TBS, 0.05% Tween 20; pH 7.4) primary antibodies were detected using Cy2-conjugated anti-mouse secondary antibodies (Dianova).

### Microscopy and data analysis

Fluorescence images were recorded using a Leica TCS-SP2 AOBS confocal laser scanning microscope (Leica Microsystems, Mannheim, Germany), equipped with a 63×/1.40 HCX PL APO oil-immersion objective, or an iMIC microscope with 100×/1.40 NA oil-immersion objective (Till Photonics, Munich, Germany). Confocal images shown are calculated maximum projections of sequential single sections processed in Adobe Photoshop (Adobe Systems). Images for the quantification of telomere clustering were taken using the iMIC and the Live Acquisition software package. 3D reconstruction, analysis and quantification of telomere attachment and clustering were conducted using the three-dimensional reconstruction tool of ImageJ (version 1.42q; http://rsbweb.nih.gov/ij).

### Statistical analysis

All statistics shown were calculated using R (version 2.10.1; http://www.r-project.org), Microsoft Office Excel 2007 or StatSoft STATISTICA 10. *P*-values were generated using Pearson's Chi^2^ test or Mann-Whitney U-test with the significance level set <0.05.

## Supporting Information

Figure S1 Mature sperm are absent in the lamin C2-deficient strain. (A) Histological sections of epididymis from wildtype and lamin C2-deficient mice showing the complete absence of mature sperm in the knockout. Scale bar 100 µm. (B) In situ labelling of apoptotic cells in testis sections from wild-type and *lamin C2^−/−^* animals using the TUNEL revealed significantly increased cell death in lamin C2-deficient mice. Scale bar 50 µm.
(TIF)

Figure S2 Male germ cells deficient for lamin C2 are more severely affected by synaptic defects than female cells when compared on single cell level. The number of affected chromosomes per cell is plotted on the x-axis. The portion of cells displaying defects is plotted on the y-axis. While the number of chromosomes that show incomplete synapsis is rarely more than three chromosomes per cell in lamin C2-deficient females (grey bars, n = 56), *lamin C2^−/−^* males frequently have up to ten chromosomes per spermatocyte affected by defective synapsis (black bars; n = 81).
(TIF)

Table S1 Primer list.
(DOC)

Video S1 Representative three-dimensional reconstruction of a *lamin C2^−/−^* spermatocyte. Immunofluorescence staining using an anti-SUN1 antibody in co-localisation with TeloFISH labelled telomeres on structurally preserved nuclei from *lamin C2^−/−^* testis. Three-dimensional reconstructions show peripheral localisation of telomeres coinciding with SUN1 signals demonstrating intact telomere attachment even in the absence of lamin C2. Scale bar 5 µm.
(MOV)

Video S2 Representative three-dimensional reconstructions of d 14 pp spermatocytes. Three-dimensional reconstructions of structurally preserved spermatocytes from 14 day old wildtype and *lamin C2^−/−^* mice stained with anti-SYCP3 and anti-SUN1. Note that at day 14 pp a significant number of *lamin C2^−/−^* spermatocytes show characteristically clustered telomeres, while in the wildtype most telomeres are evenly dispersed at the NE. Scale bars 5 µm.
(MOV)

## References

[1] Alsheimer M (2009) The dance floor of meiosis: evolutionary conservation of nuclear envelope attachment and dynamics of meiotic telomeres. Genome Dyn 5: 81–93. 18948709 10.1159/000166621

[2] Dechat T, Adam SA, Taimen P, Shimi T, Goldman RD (2010) Nuclear lamins. Cold Spring Harb Perspect Biol 2: a000547. 20826548 10.1101/cshperspect.a000547 PMC2964183

[3] Gruenbaum Y, Margalit A, Goldman RD, Shumaker DK, Wilson KL (2005) The nuclear lamina comes of age. Nat Rev Mol Cell Biol 6: 21–31. 15688064 10.1038/nrm1550

[4] Worman HJ, Ostlund C, Wang Y (2010) Diseases of the nuclear envelope. Cold Spring Harb Perspect Biol 2: a000760. 20182615 10.1101/cshperspect.a000760 PMC2828284

[5] Vester B, Smith A, Krohne G, Benavente R (1993) Presence of a nuclear lamina in pachytene spermatocytes of the rat. J Cell Sci 104 Pt 2: 557–563. 8505378 10.1242/jcs.104.2.557

[6] Furukawa K, Inagaki H, Hotta Y (1994) Identification and cloning of an mRNA coding for a germ cell-specific A-type lamin in mice. Exp Cell Res 212: 426–430. 8187835 10.1006/excr.1994.1164

[7] Alsheimer M, Benavente R (1996) Change of karyoskeleton during mammalian spermatogenesis: expression pattern of nuclear lamin C2 and its regulation. Exp Cell Res 228: 181–188. 8912709 10.1006/excr.1996.0315

[8] Alsheimer M, von Glasenapp E, Schnolzer M, Heid H, Benavente R (2000) Meiotic lamin C2: the unique amino-terminal hexapeptide GNAEGR is essential for nuclear envelope association. Proc Natl Acad Sci U S A 97: 13120–13125. 11078531 10.1073/pnas.240466597 PMC27188

[9] Alsheimer M, Jahn D, Schramm S, Benavente R (2011) Nuclear Lamins in Mammalian Spermatogenesis. In: Rousseaux S, Khochbin S, editors. Epigenetics and Human Reproduction: Springer, New York. pp. 279–288.

[10] Krohne G (1998) Lamin assembly in vivo. Subcell biochem 31: 563–586. 9932506

[11] Stuurman N, Heins S, Aebi U (1998) Nuclear lamins: their structure, assembly, and interactions. J Struct Biol 122: 42–66. 9724605 10.1006/jsbi.1998.3987

[12] Jahn D, Schramm S, Benavente R, Alsheimer M (2010) Dynamic properties of meiosis-specific lamin C2 and its impact on nuclear envelope integrity. Nucleus 1: 273–283. 21327075 10.4161/nucl.1.3.11800 PMC3027034

[13] Alsheimer M, von Glasenapp E, Hock R, Benavente R (1999) Architecture of the nuclear periphery of rat pachytene spermatocytes: distribution of nuclear envelope proteins in relation to synaptonemal complex attachment sites. Mol Biol Cell 10: 1235–1245. 10198069 10.1091/mbc.10.4.1235 PMC25260

[14] de La Roche Saint-Andre C (2008) Alternative ends: telomeres and meiosis. Biochimie 90: 181–189. 17905509 10.1016/j.biochi.2007.08.010

[15] Sullivan T, Escalante-Alcalde D, Bhatt H, Anver M, Bhat N, et al (1999) Loss of A-type lamin expression compromises nuclear envelope integrity leading to muscular dystrophy. J Cell Biol 147: 913–920. 10579712 10.1083/jcb.147.5.913 PMC2169344

[16] Alsheimer M, Liebe B, Sewell L, Stewart CL, Scherthan H, et al (2004) Disruption of spermatogenesis in mice lacking A-type lamins. J Cell Sci 117: 1173–1178. 14996939 10.1242/jcs.00975

[17] Jahn D, Schramm S, Schnolzer M, Heilmann CJ, de Koster CG, et al (2012) A truncated lamin A in the Lmna (−/−) mouse line: Implications for the understanding of laminopathies. Nucleus 3: 463–474. 22895093 10.4161/nucl.21676 PMC3474667

[18] Nakajima N, Abe K (1995) Genomic structure of the mouse A-type lamin gene locus encoding somatic and germ cell-specific lamins. FEBS Lett 365: 108–114. 7781761 10.1016/0014-5793(95)00453-g

[19] Zickler D, Kleckner N (1998) The leptotene-zygotene transition of meiosis. Annu Rev Genet 32: 619–697. 9928494 10.1146/annurev.genet.32.1.619

[20] Scherthan H, Weich S, Schwegler H, Heyting C, Harle M, et al (1996) Centromere and telomere movements during early meiotic prophase of mouse and man are associated with the onset of chromosome pairing. J Cell Biol 134: 1109–1125. 8794855 10.1083/jcb.134.5.1109 PMC2120985

[21] Bass HW (2003) Telomere dynamics unique to meiotic prophase: formation and significance of the bouquet. Cell Mol Life Sci 60: 2319–2324. 14625678 10.1007/s00018-003-3312-4 PMC11138934

[22] Chikashige Y, Tsutsumi C, Yamane M, Okamasa K, Haraguchi T, et al (2006) Meiotic proteins bqt1 and bqt2 tether telomeres to form the bouquet arrangement of chromosomes. Cell 125: 59–69. 16615890 10.1016/j.cell.2006.01.048

[23] Chikashige Y, Yamane M, Okamasa K, Tsutsumi C, Kojidani T, et al (2009) Membrane proteins Bqt3 and -4 anchor telomeres to the nuclear envelope to ensure chromosomal bouquet formation. J Cell Biol 187: 413–427. 19948484 10.1083/jcb.200902122 PMC2779253

[24] Hiraoka Y, Dernburg AF (2009) The SUN rises on meiotic chromosome dynamics. Dev Cell 17: 598–605. 19922865 10.1016/j.devcel.2009.10.014

[25] Starr DA, Fridolfsson HN (2010) Interactions between nuclei and the cytoskeleton are mediated by SUN-KASH nuclear-envelope bridges. Annu Rev Cell Dev Biol 26: 421–444. 20507227 10.1146/annurev-cellbio-100109-104037 PMC4053175

[26] Crisp M, Liu Q, Roux K, Rattner JB, Shanahan C, et al (2006) Coupling of the nucleus and cytoplasm: role of the LINC complex. J Cell Biol 172: 41–53. 16380439 10.1083/jcb.200509124 PMC2063530

[27] Schmitt J, Benavente R, Hodzic D, Hoog C, Stewart CL, et al (2007) Transmembrane protein Sun2 is involved in tethering mammalian meiotic telomeres to the nuclear envelope. Proc Natl Acad Sci U S A 104: 7426–7431. 17452644 10.1073/pnas.0609198104 PMC1863442

[28] Ding X, Xu R, Yu J, Xu T, Zhuang Y, et al (2007) SUN1 is required for telomere attachment to nuclear envelope and gametogenesis in mice. Dev Cell 12: 863–872. 17543860 10.1016/j.devcel.2007.03.018

[29] Chi YH, Cheng LI, Myers T, Ward JM, Williams E, et al (2009) Requirement for Sun1 in the expression of meiotic reproductive genes and piRNA. Development 136: 965–973. 19211677 10.1242/dev.029868 PMC2727561

[30] Adelfalk C, Janschek J, Revenkova E, Blei C, Liebe B, et al (2009) Cohesin SMC1beta protects telomeres in meiocytes. J Cell Biol 187: 185–199. 19841137 10.1083/jcb.200808016 PMC2768837

[31] Koszul R, Kleckner N (2009) Dynamic chromosome movements during meiosis: a way to eliminate unwanted connections? Trends Cell Biol 19: 716–724. 19854056 10.1016/j.tcb.2009.09.007 PMC2787882

[32] Goetz P, Chandley AC, Speed RM (1984) Morphological and temporal sequence of meiotic prophase development at puberty in the male mouse. J Cell Sci 65: 249–263. 6538881 10.1242/jcs.65.1.249

[33] Niwa O, Shimanuki M, Miki F (2000) Telomere-led bouquet formation facilitates homologous chromosome pairing and restricts ectopic interaction in fission yeast meiosis. EMBO J 19: 3831–3840. 10899136 10.1093/emboj/19.14.3831 PMC313979

[34] Ding DQ, Yamamoto A, Haraguchi T, Hiraoka Y (2004) Dynamics of homologous chromosome pairing during meiotic prophase in fission yeast. Dev Cell 6: 329–341. 15030757 10.1016/s1534-5807(04)00059-0

[35] Wanat JJ, Kim KP, Koszul R, Zanders S, Weiner B, et al (2008) Csm4, in collaboration with Ndj1, mediates telomere-led chromosome dynamics and recombination during yeast meiosis. PLoS Genet 4: e1000188 doi:10.1371/journal.pgen.1000188. 18818741 10.1371/journal.pgen.1000188 PMC2533701

[36] Conrad MN, Lee CY, Chao G, Shinohara M, Kosaka H, et al (2008) Rapid telomere movement in meiotic prophase is promoted by NDJ1, MPS3, and CSM4 and is modulated by recombination. Cell 133: 1175–1187. 18585352 10.1016/j.cell.2008.04.047

[37] Inagaki A, Schoenmakers S, Baarends WM (2010) DNA double strand break repair, chromosome synapsis and transcriptional silencing in meiosis. Epigenetics 5: 255–266. 20364103 10.4161/epi.5.4.11518

[38] Liebe B, Petukhova G, Barchi M, Bellani M, Braselmann H, et al (2006) Mutations that affect meiosis in male mice influence the dynamics of the mid-preleptotene and bouquet stages. Exp Cell Res 312: 3768–3781. 17010969 10.1016/j.yexcr.2006.07.019

[39] Mahadevaiah SK, Turner JM, Baudat F, Rogakou EP, de Boer P, et al (2001) Recombinational DNA double-strand breaks in mice precede synapsis. Nat Genet 27: 271–276. 11242108 10.1038/85830

[40] Baker SM, Plug AW, Prolla TA, Bronner CE, Harris AC, et al (1996) Involvement of mouse Mlh1 in DNA mismatch repair and meiotic crossing over. Nat Genet 13: 336–342. 8673133 10.1038/ng0796-336

[41] de Rooij DG, de Boer P (2003) Specific arrests of spermatogenesis in genetically modified and mutant mice. Cytogenet Genome Res 103: 267–276. 15051947 10.1159/000076812

[42] Kierszenbaum AL, Rivkin E, Tres LL (2003) The actin-based motor myosin Va is a component of the acroplaxome, an acrosome-nuclear envelope junctional plate, and of manchette-associated vesicles. Cytogenet Genome Res 103: 337–344. 15051957 10.1159/000076822

[43] Hunt PA, Hassold TJ (2002) Sex matters in meiosis. Science 296: 2181–2183. 12077403 10.1126/science.1071907

[44] Daniel K, Lange J, Hached K, Fu J, Anastassiadis K, et al (2011) Meiotic homologue alignment and its quality surveillance are controlled by mouse HORMAD1. Nat Cell Biol 13: 599–U232. 21478856 10.1038/ncb2213 PMC3087846

[45] Woods LM, Hodges CA, Baart E, Baker SM, Liskay M, et al (1999) Chromosomal influence on meiotic spindle assembly: abnormal meiosis I in female Mlh1 mutant mice. J Cell Biol 145: 1395–1406. 10385520 10.1083/jcb.145.7.1395 PMC2133173

[46] Yuan L, Liu JG, Hoja MR, Wilbertz J, Nordqvist K, et al (2002) Female germ cell aneuploidy and embryo death in mice lacking the meiosis-specific protein SCP3. Science 296: 1115–1118. 12004129 10.1126/science.1070594

[47] Ortega S, Prieto I, Odajima J, Martin A, Dubus P, et al (2003) Cyclin-dependent kinase 2 is essential for meiosis but not for mitotic cell division in mice. Nat Genet 35: 25–31. 12923533 10.1038/ng1232

[48] Schramm S, Fraune J, Naumann R, Hernandez-Hernandez A, Hoog C, et al (2011) A novel mouse synaptonemal complex protein is essential for loading of central element proteins, recombination, and fertility. PLoS Genet 7: e1002088 doi:10.1371/journal.pgen.1002088. 21637789 10.1371/journal.pgen.1002088 PMC3102746

[49] Scherthan H, Jerratsch M, Li B, Smith S, Hulten M, et al (2000) Mammalian meiotic telomeres: protein composition and redistribution in relation to nuclear pores. Mol Biol Cell 11: 4189–4203. 11102517 10.1091/mbc.11.12.4189 PMC15066

[50] de Boer E, Lhuissier FGP, Heyting C (2009) Cytological Analysis of Interference in Mouse Meiosis. In: Keeney S, editor. Meiosis, Vol 2: Cytological Methods. pp. 355–382.

[51] Winkel K, Alsheimer M, Ollinger R, Benavente R (2009) Protein SYCP2 provides a link between transverse filaments and lateral elements of mammalian synaptonemal complexes. Chromosoma 118: 259–267. 19034475 10.1007/s00412-008-0194-0

[52] Gob E, Schmitt J, Benavente R, Alsheimer M (2010) Mammalian sperm head formation involves different polarization of two novel LINC complexes. PLoS ONE 5: e12072 doi:10.1371/journal.pone.0012072. 20711465 10.1371/journal.pone.0012072 PMC2919408

[53] Alsheimer M, Drewes T, Schutz W, Benavente R (2005) The cancer/testis antigen CAGE-1 is a component of the acrosome of spermatids and spermatozoa. Eur J Cell Biol 84: 445–452. 15819420 10.1016/j.ejcb.2004.11.003

