# Supplementary figures and images for: The Meiotic Nuclear Lamina Regulates Chromosome Dynamics and Promotes Efficient Homologous Recombination in the Mouse
Source: PLoS Genet. 2013 Jan 31;9(1):e1003261. doi: 10.1371/journal.pgen.1003261 (PMC3561109; doi:10.1371/journal.pgen.1003261)

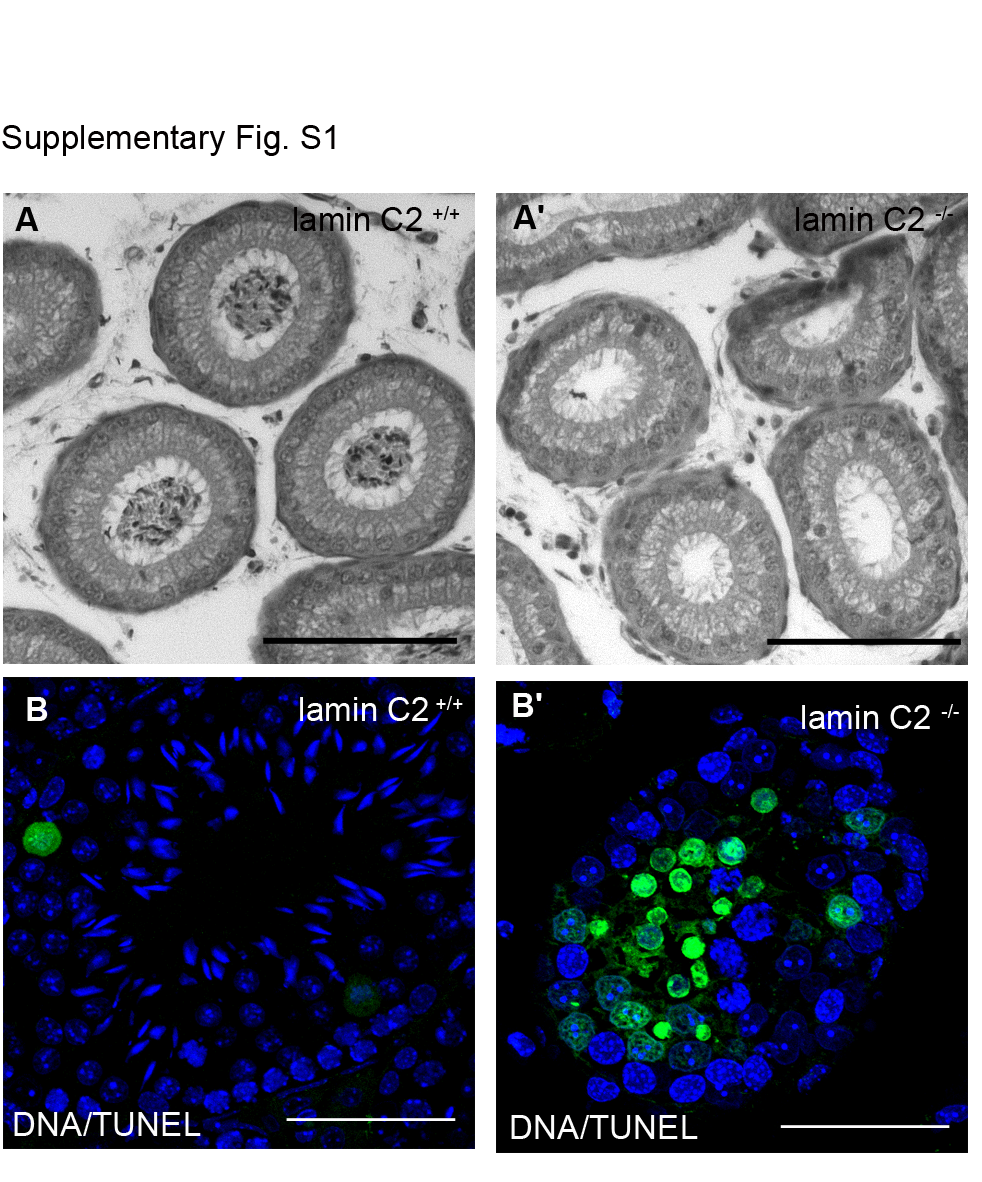

Supplement: Figure S1 — Mature sperm are absent in the lamin C2-deficient strain. (A) Histological sections of epididymis from wildtype and lamin C2-deficient mice showing the complete absence of mature sperm in the knockout. Scale bar 100 µm. (B) In situ labelling of apoptotic cells in testis sections from wild-type and lamin C2−/− animals using the TUNEL revealed significantly increased cell death in lamin C2-deficient mice. Scale bar 50 µm. (TIF) [file pgen.1003261.s001.tif]

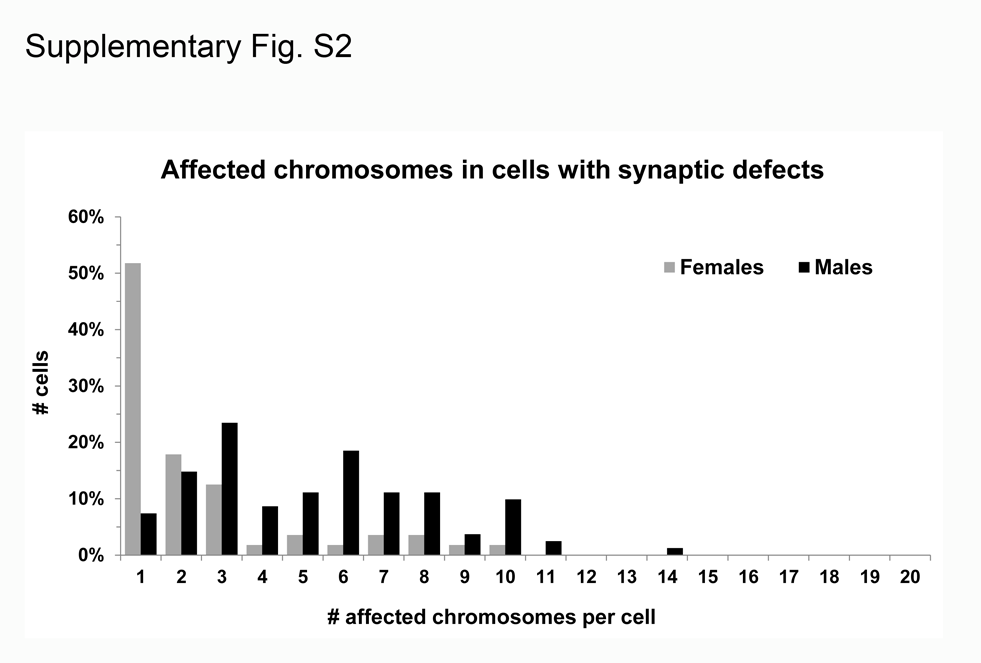

Supplement: Figure S2 — Male germ cells deficient for lamin C2 are more severely affected by synaptic defects than female cells when compared on single cell level. The number of affected chromosomes per cell is plotted on the x-axis. The portion of cells displaying defects is plotted on the y-axis. While the number of chromosomes that show incomplete synapsis is rarely more than three chromosomes per cell in lamin C2-deficient females (grey bars, n = 56), lamin C2−/− males frequently have up to ten chromosomes per spermatocyte affected by defective synapsis (black bars; n = 81). (TIF) [file pgen.1003261.s002.tif]
