# Supplementary material for: The Meiotic Nuclear Lamina Regulates Chromosome Dynamics and Promotes Efficient Homologous Recombination in the Mouse
Source: PLoS Genet. 2013 Jan 31;9(1):e1003261. doi: 10.1371/journal.pgen.1003261 (PMC3561109; doi:10.1371/journal.pgen.1003261)
Supplement: Table S1 — Primer list. (DOC) [file pgen.1003261.s003.doc]

**Supplementary Table 1**

|  | **forward primer 5’-3’** | **reverse primer 3’-5’** | **annealing (°C)** | **cycles** |
| --- | --- | --- | --- | --- |
| Lamin C2 F1 (1949 bp) | GGTACCTCTCTCAGTAAGATGTTCCACTAACAAAGTGACA | GTCGACCACACAGTAAGTGCCACGGTCTGG | 63 | 35 |
| Lamin C2 F2 (5044 bp) | GAATTCCGCCTCTAGTGCCCAAG | GAATTCGAGATAAGGTCTGGTAGTCTAGGCTGGTCTC | 60 | 35 |
| Lamin C2 southern probe | GCCATTCCCCACTAGGGTCC | CAGGTCTTGTAACTTCTCCAGTGTCTC | 54 | 35 |
| Lamin C2 WT | CACCAGAGCCCTACCAGACTCTC | AAAGCTTACACCAGCTCTGGATTC | 60 | 35 |
| Lamin C2 KO | GAAGTGTATGTGGAACAGAGGCTG | GCTTCCTCGTGCTTTACGGTATC | 60 | 35 |
| Lamin A/C  RT-PCR | CATATCGAGACCCCGTCACAGCGGC | TGGTCCTCATGCTGGGCCCGC | 64 | 25 |
| Lamin C2  RT-PCR | CATATGGGGAACGCGGAGGGCC | TGGTCCTCATGCTGGGCCCGC | 65 | 25 |
| note: PCR-amplifications were done using *Phusion****^TM^*** High-Fidelity DNA Polymerase (Finnzymes, Espoo, Finland) according to the manufacturer’s protocol. | | | | |
